# Padé approximant related to asymptotics for the gamma function

**DOI:** 10.1186/s13660-017-1315-1

**Published:** 2017-02-28

**Authors:** Xin Li, Chao-Ping Chen

**Affiliations:** 1 Department of Mathematics, East China Normal University, 500 Dongchuan Road, Shanghai, 200241 People’s Republic of China; 2 School of Mathematics and Informatics, Henan Polytechnic University, Jiaozuo, Henan 454000 China

**Keywords:** 33B15, 41A60, 26D15, gamma function, psi function, inequality, approximation

## Abstract

Based on the Padé approximation method, we determine the coefficients $a_{j}$ and $b_{j}$ ($1\leq j\leq k$) such that

$$ \frac{\Gamma(x+1)}{\sqrt{2\pi x}(x/e)^{x}}=\frac{x^{k}+a_{1}x^{k-1} +\cdots+a_{k}}{x^{k}+b_{1}x^{k-1}+\cdots+b_{k}}+O \biggl(\frac {1}{x^{2k+1}} \biggr),\quad x\to \infty, $$

where $k\geq1$ is any given integer. Based on the obtained result, we establish new bounds for the gamma function.

## Introduction

Stirling’s formula

1.1 $$\begin{aligned} n!\sim\sqrt{2\pi n} \biggl(\frac{n}{e} \biggr)^{n},\quad n \in \mathbb{N}:=\{1,2,\ldots\} \end{aligned}$$

has many applications in statistical physics, probability theory and number theory. Actually, it was first discovered in 1733 by the French mathematician Abraham de Moivre (1667-1754) in the form

$$\begin{aligned} n!\sim\mbox{constant}\cdot\sqrt{n}(n/e)^{n} \end{aligned}$$

when he was studying the Gaussian distribution and the central limit theorem. Afterwards, the Scottish mathematician James Stirling (1692-1770) found the missing constant $\sqrt{2\pi}$ when he was trying to give the normal approximation of the binomial distribution.

Stirling’s series for the gamma function is given (see [1], p.257, Eq. (6.1.40)) by

1.2 $$\begin{aligned} \Gamma(x+1)&\sim\sqrt{2\pi x} \biggl(\frac{x}{e} \biggr)^{x}\exp \Biggl(\sum_{m=1}^{\infty} \frac {B_{2m}}{2m(2m-1)x^{2m-1}} \Biggr) \end{aligned}$$

as $x\to\infty$, where $B_{n}$ $(n \in\mathbb{N}_{0}:=\mathbb{N} \cup\{0\} )$ are the Bernoulli numbers defined by the following generating function:

1.3 $$ \frac{z}{e^{z}-1}=\sum_{n=0}^{\infty} B_{n} \frac{z^{n}}{n!}, \quad|z|< 2\pi. $$

The following asymptotic formula is due to Laplace:

1.4 $$\begin{aligned} \Gamma(x+1)&\sim\sqrt{2\pi x} \biggl(\frac{x}{e} \biggr)^{x} \biggl(1+\frac{1}{12x}+\frac {1}{288x^{2}}- \frac{139}{51{,}840x^{3}}-\frac{571}{2{,}488{,}320x^{4}}+\cdots \biggr) \end{aligned}$$

as $x\to\infty$ (see [1], p.257, Eq. (6.1.37)).

The expression (1.4) is sometimes incorrectly called Stirling’s series (see [2], pp.2-3). Stirling’s formula is in fact the first approximation to the asymptotic formula (1.4). Stirling’s formula has attracted much interest of many mathematicians and has motivated a large number of research papers concerning various generalizations and improvements (see [3–54] and the references cited therein). It is interesting to note that the aforementioned mathematicians represent many nationalities. So the topic is of interest for mathematicians from diverse cultural background.

Using the Maple software, we find, as $x\to\infty$,

1.5 $$\begin{aligned} \frac{\Gamma(x+1)}{\sqrt{2\pi x}(x/e)^{x}}=\frac{x+\frac{1}{24}}{x-\frac {1}{24}}+O \biggl(\frac{1}{x^{3}} \biggr) \end{aligned}$$

and

1.6 $$\begin{aligned} \frac{\Gamma(x+1)}{\sqrt{2\pi x}(x/e)^{x}}=\frac{x^{2}+\frac{1}{24}x+\frac {293}{8{,}640}}{x^{2}-\frac{1}{24}x+\frac{293}{8{,}640}}+O \biggl(\frac {1}{x^{5}} \biggr). \end{aligned}$$

Based on the Padé approximation method, in this paper we develop the approximation formulas (1.5) and (1.6) to produce a general result. More precisely, we determine the coefficients $a_{j}$ and $b_{j}$ ($1\leq j\leq k$) such that

$$ \frac{\Gamma(x+1)}{\sqrt{2\pi x}(x/e)^{x}}=\frac{x^{k}+a_{1}x^{k-1}+\cdots +a_{k}}{x^{k}+b_{1}x^{k-1}+\cdots+b_{k}}+O \biggl(\frac{1}{x^{2k+1}} \biggr), \quad x\to \infty, $$

where $k\geq1$ is any given integer. Based on the obtained result, we establish new bounds for the gamma function.

The numerical values given in this paper have been calculated via the computer program MAPLE 13.

## Lemmas

The following lemmas are required in our present investigation.

### Lemma 2.1

[9]

*Let* *r* *be a given nonzero real number*. *The gamma function has the following asymptotic formula*:

2.1 $$\begin{aligned} \Gamma(x+1)\sim\sqrt{2\pi x} \biggl(\frac{x}{e} \biggr)^{x} \Biggl(1+\sum_{j=1}^{\infty} \frac {b_{j}}{x^{j}} \Biggr)^{1/r},\quad x\to\infty, \end{aligned}$$

*with the coefficients* $b_{j}=b_{j}(r) $ ($j=1,2,\ldots$) *given by*

2.2 $$ b_{j}=\sum_{k_{1}+2k_{2}+\cdots+jk_{j}=j} \frac{r^{k_{1}+k_{2}+\cdots +k_{j}}}{k_{1}!k_{2}!\cdots k_{j}!} \biggl(\frac{B_{2}}{1\cdot2} \biggr)^{k_{1}} \biggl( \frac{B_{3}}{2\cdot 3} \biggr)^{k_{2}}\cdots \biggl(\frac{B_{j+1}}{j(j+1)} \biggr)^{k_{j}}, $$

*where* $B_{n}$ $(n \in\mathbb{N}_{0}:=\mathbb{N} \cup\{0\} )$ *are the Bernoulli numbers defined in* (1.3), *summed over all nonnegative integers* $k_{j}$ *satisfying the equation* $k_{1}+2k_{2}+\cdots+jk_{j}=j$.

Laplace formula (1.4) can be rewritten as

2.3 $$\begin{aligned} \Gamma(x+1)&\sim\sqrt{2\pi x} \biggl(\frac{x}{e} \biggr)^{x} \Biggl(\sum_{j=0}^{\infty} \frac {c_{j}}{x^{j}} \Biggr),\quad x \to\infty, \end{aligned}$$

with the coefficients $c_{j}$ given by

2.4 $$ \begin{aligned} &c_{0}=1, \\ &c_{j}=\sum _{k_{1}+2k_{2}+\cdots+{j}k_{j}={j}}\frac {1}{k_{1}!k_{2}!\cdots k_{j}!} \biggl(\frac{B_{2}}{1\cdot2} \biggr)^{k_{1}} \biggl(\frac{B_{3}}{2\cdot 3} \biggr)^{k_{2}}\cdots \biggl(\frac{B_{{j}+1}}{{j}({j}+1)} \biggr)^{k_{j}} \quad\mbox{for } j\geq1. \end{aligned} $$

### Lemma 2.2

[55], Theorem 8

*Let* $n \geq0$ *be an integer*. *The functions*

$$ F_{n}(x)=\ln\Gamma(x)- \biggl(x-\frac{1}{2} \biggr)\ln x+x-\frac{1}{2}\ln(2\pi)-\sum_{j=1}^{2n} \frac{B_{2j}}{2j(2j-1)x^{2j-1}} $$

*and*

$$ G_{n}(x)=-\ln\Gamma(x)+ \biggl(x-\frac{1}{2} \biggr)\ln x-x+\frac{1}{2}\ln(2\pi)+\sum_{j=1}^{2n+1} \frac{B_{2j}}{2j(2j-1)x^{2j-1}} $$

*are completely monotonic on* $(0, \infty)$. *Here* $B_{n}$ $(n \in\mathbb{N}_{0}:=\mathbb{N}\cup\{0\})$ *are the Bernoulli numbers*.

### Remark 2.1

Lemma 2.2 can be stated as follows: for every $m\in\mathbb{N}_{0}$, the function

$$ R_{m}(x)=(-1)^{m} \Biggl[\ln\Gamma(x)- \biggl(x-\frac{1}{2} \biggr)\ln x+x-\ln \sqrt{2\pi}-\sum _{j=1}^{m}\frac{B_{2j}}{2j(2j-1)x^{2j-1}} \Biggr] $$

is completely monotonic on $(0, \infty)$.

In 2006, Koumandos [56] presented a simpler proof of complete monotonicity of the functions $R_{m}(x)$. In 2009, Koumandos and Pedersen [57], Theorem 2.1, strengthened this result.

From $F_{n}'(x)<0$ and $G_{n}'(x)<0$ for $x>0$, we obtain

2.5 $$ \sum_{j=1}^{2n} \frac{B_{2j}}{2jx^{2j}}< \ln x-\psi(x)-\frac{1}{2x}< \sum _{j=1}^{2n+1}\frac{B_{2j}}{2jx^{2j}}, \quad x>0, $$

where $\psi(x)=\Gamma'(x)/\Gamma(x)$ is the psi (or digamma) function. Noting that

$$ \psi(x+1)=\psi(x)+\frac{1}{x} $$

holds, we obtain from (2.5) that for $x>0$,

2.6 $$\begin{aligned} &{-}\frac{1}{12x^{2}}+\frac{1}{120x^{4}}-\frac{1}{252x^{6}}+ \frac {1}{240x^{8}}-\frac{1}{132x^{10}}< \psi(x+1)-\ln x-\frac{1}{2x} \\ &\quad < -\frac{1}{12x^{2}}+\frac{1}{120x^{4}}-\frac {1}{252x^{6}}+\frac{1}{240x^{8}}- \frac{1}{132x^{10}}+\frac{691}{32{,}760x^{12}}. \end{aligned}$$

## Approximations to the gamma function

For our later use, we introduce Padé approximant (see [58–61]). Let *f* be a formal power series

3.1 $$\begin{aligned} f(t)=c_{0}+c_{1}t+c_{2}t^{2}+ \cdots. \end{aligned}$$

The Padé approximation of order $(p, q)$ of the function *f* is the rational function, denoted by

3.2 $$\begin{aligned}{} [p/q]_{f}(t)=\frac{\sum_{j=0}^{p}a_{j}t^{j}}{1+\sum_{j=1}^{q}b_{j}t^{j}}, \end{aligned}$$

where $p\geq0$ and $q\geq1$ are two given integers, the coefficients $a_{j}$ and $b_{j}$ are given by (see [58–60])

3.3 $$\begin{aligned} \textstyle\begin{cases} a_{0}=c_{0},\\ a_{1}=c_{0}b_{1}+c_{1},\\ a_{2}=c_{0}b_{2}+c_{1}b_{1}+c_{2}\,\ \vdots\\ a_{p} = c_{0}b_{p}+\cdots+ c_{p-1}b_{1} + c_{p},\\ 0 = c_{p+1} + c_{p}b_{1} + \cdots+ c_{p-q+1}b_{q},\\ \vdots&\\ 0 = c_{p+q} + c_{p+q-1}b_{1} + \cdots+ c_{p}b_{q}, \end{cases}\displaystyle \end{aligned}$$

and the following holds:

3.4 $$\begin{aligned}{} [p/q]_{f}(t)- f (t) = O\bigl(t^{p+q+1} \bigr). \end{aligned}$$

Thus, the first $p + q + 1$ coefficients of the series expansion of $[p/q]_{f}$ are identical to those of *f*. Moreover, we have (see [61])

3.5 $$ [p/q]_{f}(t)= \frac{\left| {\scriptsize\begin{matrix}{}t^{q}f_{p-q}(t) & t^{q-1}f_{p-q+1}(t) &\cdots &f_{p}(t) \cr c_{p-q+1} & c_{p-q+2} &\cdots &c_{p+1} \cr \vdots &\vdots &\ddots &\vdots \cr c_{p} & c_{p+1} &\cdots &c_{p+q} \end{matrix}}\right|}{\left| {\scriptsize\begin{matrix}{} t^{q} & t^{q-1} &\cdots &1 \cr c_{p-q+1} & c_{p-q+2} &\cdots &c_{p+1} \cr \vdots &\vdots &\ddots &\vdots \cr c_{p} & c_{p+1} &\cdots &c_{p+q} \end{matrix}}\right|}, $$

with $f_{n}(x) = c_{0}+ c_{1}x+ \cdots+ c_{n}x^{n}$, the *n*th partial sum of the series *f* in (3.1) ($f_{n}$ is identically zero for $n < 0$).

Let

3.6 $$\begin{aligned} f(x)=\frac{\Gamma(x+1)}{\sqrt{2\pi x}(x/e)^{x}}. \end{aligned}$$

It follows from (2.3) that, as $x\to\infty$,

3.7 $$\begin{aligned} f(x)\sim\sum_{j=0}^{\infty} \frac{c_{j}}{x^{j}}=1+\frac{1}{12x}+\frac {1}{288x^{2}}-\frac{139}{51{,}840x^{3}}- \frac{571}{2{,}488{,}320x^{4}}+\cdots, \end{aligned}$$

with the coefficients $c_{j}$ given by (2.4). In what follows, the function *f* is given in (3.6).

Based on the Padé approximation method, we now give a derivation of formula (1.5). To this end, we consider

$$\begin{aligned}{} [1/1]_{f}(x)=\frac{\sum_{j=0}^{1}a_{j}x^{-j}}{1+\sum_{j=1}^{1}b_{j}x^{-j}}. \end{aligned}$$

Noting that

3.8 $$\begin{aligned} c_{0}=1,\qquad c_{1}=\frac{1}{12},\qquad c_{2}=\frac{1}{288},\qquad c_{3}=-\frac {139}{51{,}840},\qquad c_{4}=-\frac{571}{2{,}488{,}320} \end{aligned}$$

holds, we have, by (3.3),

$$\begin{aligned} \textstyle\begin{cases} a_{0}=1,\\ a_{1}=b_{1}+\frac{1}{12},\\ 0 =\frac{1}{288}+ \frac{1}{12}b_{1}, \end{cases}\displaystyle \end{aligned}$$

that is,

$$\begin{aligned} a_{0}=1,\qquad a_{1}=\frac{1}{24},\qquad b_{1}=- \frac{1}{24}. \end{aligned}$$

We thus obtain that

3.9 $$ [1/1]_{f}(x)= \frac{1+\frac{1}{24x}}{1-\frac{1}{24x}}=\frac{x+\frac {1}{24}}{x-\frac{1}{24}}, $$

and we have, by (3.4),

$$ \frac{\Gamma(x+1)}{\sqrt{2\pi x}(x/e)^{x}}=\frac{x+\frac{1}{24}}{x-\frac {1}{24}}+O \biggl(\frac{1}{x^{3}} \biggr). $$

We now give a derivation of formula (1.6). To this end, we consider

$$\begin{aligned}{} [2/2]_{f}(x)=\frac{\sum_{j=0}^{2}a_{j}x^{-j}}{1+\sum_{j=1}^{2}b_{j}x^{-j}}. \end{aligned}$$

Noting that (3.8) holds, we have, by (3.3),

$$\begin{aligned} \textstyle\begin{cases} a_{0}=1,\\ a_{1}=b_{1}+\frac{1}{12},\\ a_{2} =b_{2}+\frac{1}{12}b_{1}+ \frac{1}{288}, \\ 0 = -\frac{139}{51{,}840}+\frac{1}{288}b_{1}+\frac{1}{12}b_{2},\\ 0 = -\frac{571}{2{,}488{,}320}-\frac{139}{51{,}840}b_{1}+\frac{1}{288}b_{2}, \end{cases}\displaystyle \end{aligned}$$

that is,

$$\begin{aligned} a_{0}=1,\qquad a_{1}=\frac{1}{24},\qquad a_{2} = \frac{293}{8{,}640},\qquad b_{1}=-\frac{1}{24},\qquad b_{2}= \frac{293}{8{,}640}. \end{aligned}$$

We thus obtain that

3.10 $$ [2/2]_{f}(x)= \frac{1+\frac{1}{24x}+\frac{293}{8{,}640x^{2}}}{1-\frac {1}{24x}+\frac{293}{8{,}640x^{2}}}=\frac{x^{2}+\frac{1}{24}x+\frac {293}{8{,}640}}{x^{2}-\frac{1}{24}x+\frac{293}{8{,}640}} , $$

and we have, by (3.4),

$$ \frac{\Gamma(x+1)}{\sqrt{2\pi x}(x/e)^{x}}=\frac{x^{2}+\frac{1}{24}x+\frac {293}{8{,}640}}{x^{2}-\frac{1}{24}x+\frac{293}{8{,}640}}+O \biggl(\frac {1}{x^{5}} \biggr). $$

From the Padé approximation method and the expansion (3.7), we now present a general result given by Theorem 3.1.

### Theorem 3.1

*The Padé approximation of order* $(p, q)$ *of the Laplace asymptotic formula of the function* $f(x)=\frac{\Gamma(x+1)}{\sqrt{2\pi x}(x/e)^{x}}$ (*at the point* $x=\infty$) *is the following rational function*:

3.11 $$\begin{aligned}{} [p/q]_{f}(x)=\frac{1+\sum_{j=1}^{p}a_{j}x^{-j}}{1+\sum_{j=1}^{q}b_{j}x^{-j}}=x^{q-p} \biggl(\frac{x^{p}+a_{1}x^{p-1}+\cdots +a_{p}}{x^{q}+b_{1}x^{q-1}+\cdots+b_{q}} \biggr), \end{aligned}$$

*where* $p\geq1$ *and* $q\geq1$ *are any given integers*, *the coefficients* $a_{j}$ *and* $b_{j}$ *are given by*

3.12 $$\begin{aligned} \textstyle\begin{cases} a_{1}=b_{1}+c_{1},\\ a_{2}=b_{2}+c_{1}b_{1}+c_{2},\\ \vdots\\ a_{p} = b_{p}+\cdots+ c_{p-1}b_{1} + c_{p},\\ 0 = c_{p+1} + c_{p}b_{1} + \cdots+ c_{p-q+1}b_{q},\\ \vdots&\\ 0 = c_{p+q} + c_{p+q-1}b_{1} + \cdots+ c_{p}b_{q}, \end{cases}\displaystyle \end{aligned}$$

*and* $c_{j}$ *is given in* (2.4), *and the following holds*:

3.13 $$\begin{aligned} f (x) -[p/q]_{f}(x) = O \biggl(\frac{1}{x^{p+q+1}} \biggr),\quad x\to \infty. \end{aligned}$$

*Moreover*, *we have*

3.14 $$ [p/q]_{f}(x)= \frac{\left| {\scriptsize\begin{matrix}{}\frac{1}{x^{q}}f_{p-q}(x) & \frac{1}{x^{q-1}}f_{p-q+1}(x) &\cdots &f_{p}(x) \cr c_{p-q+1} & c_{p-q+2} &\cdots &c_{p+1} \cr \vdots &\vdots &\ddots &\vdots \cr c_{p} & c_{p+1} &\cdots &c_{p+q} \end{matrix}}\right|}{\left| {\scriptsize\begin{matrix}{} \frac{1}{x^{q}} & \frac{1}{x^{q-1}} &\cdots &1 \cr c_{p-q+1} & c_{p-q+2} &\cdots &c_{p+1} \cr \vdots &\vdots &\ddots &\vdots \cr c_{p} & c_{p+1} &\cdots &c_{p+q} \end{matrix}}\right|}, $$

*with* $f_{n}(x)=\sum_{j=0}^{n}\frac{c_{j}}{x^{j}}$, *the* *nth partial sum of the asymptotic series* (3.7).

### Remark 3.1

Using (3.14), we can also derive (3.9) and (3.10). Indeed, we have

$$ [1/1]_{f}(x)=\frac{ \left| {\scriptsize\begin{matrix}{} \frac{1}{x}f_{0}(x) &f_{1}(x) \cr c_{1} &c_{2} \end{matrix}}\right|}{\left| {\scriptsize\begin{matrix}{} \frac{1}{x} &1 \cr c_{1} &c_{2} \end{matrix}}\right|} = \frac{\left| {\scriptsize\begin{matrix}{} \frac{1}{x} &1+\frac{1}{12x}\cr \frac{1}{12} &\frac{1}{288} \end{matrix}}\right|}{\left| {\scriptsize\begin{matrix}{} \frac{1}{x} &1 \cr \frac{1}{12} &\frac{1}{288} \end{matrix}}\right|}=\frac{x+\frac{1}{24}}{x-\frac{1}{24}} $$

and

$$\begin{aligned}{} [2/2]_{f}(x)&=\frac{ \left| {\scriptsize\begin{matrix}{} \frac{1}{x^{2}}f_{0}(x) & \frac{1}{x}f_{1}(x) &f_{2}(x) \cr c_{1} &c_{2} &c_{3} \cr c_{2} &c_{3} &c_{4} \end{matrix}}\right|}{\left| {\scriptsize\begin{matrix}{} \frac{1}{x^{2}} &\frac{1}{x} &1 \cr c_{1} &c_{2} &c_{3} \cr c_{2} &c_{3} &c_{4} \end{matrix}}\right|} = \frac{\left| {\scriptsize\begin{matrix}{} \frac{1}{x^{2}} & \frac{1}{x} (1+\frac{1}{12x} ) &1+\frac {1}{12x}+\frac{1}{288x^{2}}\cr \frac{1}{12} &\frac{1}{288} &-\frac{139}{51{,}840} \cr \frac{1}{288} &-\frac{139}{51{,}840} &-\frac{571}{2{,}488{,}320} \end{matrix}}\right|}{\left| {\scriptsize\begin{matrix}{} \frac{1}{x^{2}} &\frac{1}{x} &1 \cr \frac{1}{12} &\frac{1}{288} &-\frac{139}{51{,}840} \cr \frac{1}{288} &-\frac{139}{51{,}840} &-\frac{571}{2{,}488{,}320} \end{matrix}}\right|} =\frac{x^{2}+\frac{1}{24}x+\frac{293}{8{,}640}}{x^{2}-\frac{1}{24}x+\frac{293}{8{,}640}}. \end{aligned}$$

Setting $(p, q)=(k, k)$ in (3.13), we obtain the following corollary.

### Corollary 3.1

*As* $x\to\infty$,

3.15 $$ \frac{\Gamma(x+1)}{\sqrt{2\pi x}(x/e)^{x}}=\frac{x^{k}+a_{1}x^{k-1}+\cdots +a_{k}}{x^{k}+b_{1}x^{k-1}+\cdots+b_{k}}+O \biggl(\frac{1}{x^{2k+1}} \biggr), $$

*where* $k\geq1$ *is any given integer*, *the coefficients* $a_{j}$ *and* $b_{j}$ ($1\leq j\leq k$) *are given by*

3.16 $$\begin{aligned} \textstyle\begin{cases} a_{1}=b_{1}+c_{1},\\ a_{2}=b_{2}+c_{1}b_{1}+c_{2},\\ \vdots\\ a_{k} = b_{k}+\cdots+ c_{k-1}b_{1} + c_{k},\\ 0 = c_{k+1} + c_{k}b_{1} + \cdots+ c_{1}b_{k},\\ \vdots\\ 0 = c_{2k} + c_{2k-1}b_{1} + \cdots+ c_{k}b_{k}, \end{cases}\displaystyle \end{aligned}$$

*and* $c_{j}$ *is given in* (2.4).

Setting $k=3$ and $k=4$ in (3.15), respectively, yields

3.17 $$\begin{aligned} \frac{\Gamma(x+1)}{\sqrt{2\pi x}(x/e)^{x}}=\frac{x^{3}+\frac{1}{24}x^{2}+\frac {166{,}903}{590{,}688}x+\frac{4{,}406{,}147}{425{,}295{,}360}}{x^{3}-\frac{1}{24}x^{2}+\frac {166{,}903}{590{,}688}x-\frac{4{,}406{,}147}{425{,}295{,}360}}+O \biggl(\frac{1}{x^{7}} \biggr) \end{aligned}$$

and

3.18 $$\begin{aligned} \frac{\Gamma(x+1)}{\sqrt{2\pi x}(x/e)^{x}} =\frac{x^{4}+\frac{1}{24}x^{3}+\frac {685{,}893{,}605}{845{,}980{,}224}x^{2}+\frac{14{,}787{,}105{,}577}{456{,}829{,}320{,}960}x+\frac {2{,}749{,}505{,}046{,}083}{153{,}494{,}651{,}842{,}560}}{x^{4}-\frac{1}{24}x^{3}+\frac {685{,}893{,}605}{845{,}980{,}224}x^{2}-\frac{14{,}787{,}105{,}577}{456{,}829{,}320{,}960}x+\frac {2{,}749{,}505{,}046{,}083}{153{,}494{,}651{,}842{,}560}}+O \biggl(\frac{1}{x^{9}} \biggr). \end{aligned}$$

In view of (1.5), (1.6), (3.17) and (3.18), we pose the following conjecture.

### Conjecture 3.1

*The coefficients* $a_{j}$ *and* $b_{j}$ ($1\leq j\leq k$) *in* (3.15) *satisfy the following relation*:

3.19 $$ a_{j}=(-1)^{j}b_{j}, \quad j=1,2,\ldots, k. $$

## Inequalities for the gamma function

Formulas (3.17) and (3.18) motivate us to establish the following theorem.

### Theorem 4.1

*The following inequalities hold*:

4.1 $$\begin{aligned} U(x)< \frac{\Gamma(x+1)}{\sqrt{2\pi x}(x/e)^{x}}< V(x), \end{aligned}$$

*where*

4.2 $$\begin{aligned} U(x)=\frac{x^{4}+\frac{1}{24}x^{3}+\frac{685{,}893{,}605}{845{,}980{,}224}x^{2}+\frac {14{,}787{,}105{,}577}{456{,}829{,}320{,}960}x+\frac {2{,}749{,}505{,}046{,}083}{153{,}494{,}651{,}842{,}560}}{x^{4}-\frac{1}{24}x^{3}+\frac {685{,}893{,}605}{845{,}980{,}224}x^{2}-\frac{14{,}787{,}105{,}577}{456{,}829{,}320{,}960}x+\frac {2{,}749{,}505{,}046{,}083}{153{,}494{,}651{,}842{,}560}} \end{aligned}$$

*and*

4.3 $$\begin{aligned} V(x)=\frac{x^{3}+\frac{1}{24}x^{2}+\frac{166{,}903}{590{,}688}x+\frac {4{,}406{,}147}{425{,}295{,}360}}{x^{3}-\frac{1}{24}x^{2}+\frac{166{,}903}{590{,}688}x-\frac {4{,}406{,}147}{425{,}295{,}360}}. \end{aligned}$$

*The left*-*hand side inequality holds for* $x\geq3$, *while the right*-*hand side inequality is valid for* $x\geq2$.

### Proof

It suffices to show that

$$\begin{aligned} F(x)>0 \quad\mbox{for } x\geq3 \quad\mbox{and}\quad G(x)< 0 \quad\mbox{for } x\geq2, \end{aligned}$$

where

$$\begin{aligned} F(x)=\ln\Gamma(x+1)- \biggl(x+\frac{1}{2} \biggr)\ln x+x-\ln\sqrt{2\pi }- \ln U(x) \end{aligned}$$

and

$$\begin{aligned} G(x)=\ln\Gamma(x+1)- \biggl(x+\frac{1}{2} \biggr)\ln x+x-\ln\sqrt{2\pi }- \ln V(x). \end{aligned}$$

Differentiating $F(x)$ and applying the second inequality in (2.6) yield

$$\begin{aligned} F'(x)&=\psi(x+1)-\ln x-\frac{1}{2x}-\frac{U'(x)}{U(x)} \\ &< -\frac{1}{12x^{2}}+\frac{1}{120x^{4}}-\frac{1}{252x^{6}}+\frac {1}{240x^{8}}- \frac{1}{132x^{10}}+\frac{691}{32{,}760x^{12}}-\frac {U'(x)}{U(x)} \\ &=-\frac{P_{10}(x-3)}{720{,}720x^{12}P_{8}(x)}, \end{aligned}$$

where

$$\begin{aligned} P_{10}(x)={}&1{,}698{,}313{,}885{,}002{,}591{,}369{,}403{,}376{,}359{,}237{,}155{,}137 \\ &{}+7{,}041{,}090{,}100{,}510{,}955{,}203{,}400{,}650{,}726{,}407{,}309{,}444x \\ &{}+12{,}215{,}302{,}599{,}727{,}743{,}342{,}615{,}877{,}184{,}100{,}329{,}802x^{2} \\ &{}+12{,}025{,}928{,}200{,}234{,}176{,}519{,}514{,}968{,}711{,}811{,}967{,}964x^{3} \\ &{}+7{,}551{,}739{,}592{,}924{,}831{,}815{,}437{,}063{,}682{,}435{,}942{,}293x^{4} \\ &{}+3{,}187{,}338{,}342{,}726{,}357{,}084{,}428{,}868{,}676{,}747{,}628{,}952x^{5} \\ &{}+920{,}408{,}575{,}975{,}851{,}494{,}996{,}412{,}447{,}435{,}781{,}084x^{6} \\ &{}+180{,}133{,}255{,}608{,}389{,}118{,}267{,}365{,}601{,}710{,}648{,}784x^{7} \\ &{}+22{,}910{,}271{,}532{,}985{,}226{,}283{,}927{,}122{,}357{,}066{,}246x^{8} \\ &{}+1{,}711{,}635{,}468{,}441{,}001{,}446{,}976{,}320{,}994{,}717{,}320x^{9} \\ &{}+57{,}054{,}515{,}614{,}700{,}048{,}232{,}544{,}033{,}157{,}244x^{10} \end{aligned}$$

and

$$\begin{aligned} P_{8}(x)={}&\bigl(153{,}494{,}651{,}842{,}560x^{4}+6{,}395{,}610{,}493{,}440x^{3}\\ &{}+124{,}448{,}535{,}691{,}200x^{2}+4{,}968{,}467{,}473{,}872x \\ &{} +2{,}749{,}505{,}046{,}083\bigr) \bigl(153{,}494{,}651{,}842{,}560x^{4}\\ &{}-6{,}395{,}610{,}493{,}440x^{3}+124{,}448{,}535{,}691{,}200x^{2}\\ &{} -4{,}968{,}467{,}473{,}872x+2{,}749{,}505{,}046{,}083\bigr). \end{aligned}$$

Hence, $F'(x)<0$ for $x\geq3$, and we have

$$\begin{aligned} F(x)>\lim_{t\to\infty}F(t)=0 \quad\mbox{for } x\geq3. \end{aligned}$$

Differentiating $G(x)$ and applying the first inequality in (2.6) yield

$$\begin{aligned}[b] G'(x)&=\psi(x+1)-\ln x-\frac{1}{2x}-\frac{V'(x)}{V(x)} \\ &>-\frac{1}{12x^{2}}+\frac{1}{120x^{4}}-\frac{1}{252x^{6}}+\frac {1}{240x^{8}}- \frac{1}{132x^{10}}-\frac{V'(x)}{V(x)} \\ &=\frac{Q_{8}(x-2)}{55{,}440x^{10}Q_{6}(x)}, \end{aligned} $$

where

$$\begin{aligned} Q_{8}(x)={}&2{,}456{,}573{,}428{,}493{,}290{,}077{,}832 +14{,}719{,}278{,}306{,}954{,}453{,}533{,}828x\\ &{}+32{,}394{,}299{,}960{,}322{,}640{,}776{,}801x^{2} \\ &{}+37{,}478{,}643{,}384{,}199{,}534{,}772{,}000x^{3}+25{,}805{,}343{,}259{,}499{,}481{,}612{,}340x^{4} \\ &{}+11{,}004{,}898{,}939{,}796{,}249{,}295{,}384x^{5}+2{,}862{,}385{,}365{,}338{,}807{,}176{,}962x^{6} \\ &{}+416{,}852{,}240{,}076{,}239{,}943{,}360x^{7}+26{,}053{,}265{,}004{,}764{,}996{,}460x^{8} \end{aligned}$$

and

$$\begin{aligned} Q_{6}(x)={}&\bigl(425{,}295{,}360x^{3}+17{,}720{,}640x^{2}+120{,}170{,}160x+4{,}406{,}147 \bigr) \\ &{} \times\bigl(425{,}295{,}360x^{3}-17{,}720{,}640x^{2}+120{,}170{,}160x-4{,}406{,}147 \bigr). \end{aligned}$$

Hence, $G'(x)>0$ for $x\geq2$, and we have

$$\begin{aligned} G(x)< \lim_{t\to\infty}G(t)=0 \quad\mbox{for } x\geq2. \end{aligned}$$

The proof is complete. □

### Remark 4.1

Following the same method as the one used in the proof of Theorem 4.1, we can prove the double inequality

4.4 $$\begin{aligned} \frac{x^{2}+\frac{1}{24}x+\frac{293}{8{,}640}}{x^{2}-\frac{1}{24}x+\frac {293}{8{,}640}}< \frac{\Gamma(x+1)}{\sqrt{2\pi x}(x/e)^{x}}< \frac{x+\frac {1}{24}}{x-\frac{1}{24}} \end{aligned}$$

for $x\geq2$. We here omit it. Some computer experiments indicate that inequalities (4.1) and (4.4) are valid for $x\geq1$.

In view of (4.1) and (4.4), we pose the following conjecture.

### Conjecture 4.1

*If* *k* *is odd*, *then for* $x\geq1$,

4.5 $$ \frac{\Gamma(x+1)}{\sqrt{2\pi x}(x/e)^{x}}< \frac{x^{k}+a_{1}x^{k-1}+\cdots +a_{k}}{x^{k}+b_{1}x^{k-1}+\cdots+b_{k}}, $$

*where the coefficients* $a_{j}$ *and* $b_{j}$ ($1\leq j \leq k$) *are determined in* (3.16). *If* *k* *is even*, *then inequality* (4.5) *is reversed*.

## Comparison

In 2011, Mortici [47] showed by numerical computations that his formula

5.1 $$ \begin{aligned} n!\sim\sqrt{2\pi n} \biggl( \frac{n}{e} \biggr)^{n}\exp \biggl(\frac{1}{12n+\frac{2}{5n}} \biggr)= \mu_{n} \end{aligned} $$

is much stronger than other known formulas such as:

5.2 $$\begin{aligned} &n!\sim\sqrt{2\pi} \biggl(\frac{n+1/2}{e} \biggr)^{n+1/2}= \beta_{n}\quad (\mbox{Burnside [8]}), \end{aligned}$$

5.3 $$\begin{aligned} &n!\sim\frac{\sqrt{2\pi}e^{-n}n^{n+1}}{\sqrt{n-1/6}}=\delta_{n}\quad (\mbox{Batir [4]}), \end{aligned}$$

5.4 $$\begin{aligned} &n!\sim\sqrt{2\pi \biggl(n+\frac{1}{6} \biggr)} \biggl(\frac{n}{e} \biggr)^{n}=\gamma_{n}\quad(\mbox{Gosper [19]}), \end{aligned}$$

5.5 $$\begin{aligned} &n!\sim\sqrt{\pi} \biggl(\frac{n}{e} \biggr)^{n} \biggl(8n^{3}+4n^{2}+n+\frac {1}{30} \biggr)^{1/6}=\rho_{n}\quad (\mbox{Ramanujan [62], p.339}). \end{aligned}$$

In 2012, Mahmoud et al. [31] showed numerically that their formula

5.6 $$ n!\sim\sqrt{2\pi n} \biggl( \frac{n}{e} \biggr)^{n}\exp \biggl(\frac{1}{20n}+ \frac{1}{30}\zeta(2, n+1/2) \biggr)=\lambda_{n} $$

has a superiority over Mortici’s formula (5.1). Here $\zeta(s,a)$ denotes the Hurwitz (or generalized) zeta function defined by

$$ \zeta(s, a):=\sum_{k=0}^{\infty} \frac{1}{(k+a)^{s}} \quad\bigl(\Re(s)>1; a\notin Z_{0}^{-}\bigr), $$

$Z_{0}^{-}$ being the set of nonpositive integers.

From (3.17) and (3.18), we obtain

5.7 $$\begin{aligned} n!\sim\sqrt{2\pi n} \biggl(\frac{n}{e} \biggr)^{n} \frac{n^{3}+\frac {1}{24}n^{2}+\frac{166{,}903}{590{,}688}n+\frac{4{,}406{,}147}{425{,}295{,}360}}{n^{3}-\frac {1}{24}n^{2}+\frac{166{,}903}{590{,}688}n-\frac{4{,}406{,}147}{425{,}295{,}360}}=U_{n} \end{aligned}$$

and

5.8 $$\begin{aligned} n!&\sim\sqrt{2\pi n} \biggl(\frac{n}{e} \biggr)^{n} \frac{n^{4}+\frac {1}{24}n^{3}+\frac{685{,}893{,}605}{845{,}980{,}224}n^{2}+\frac {14{,}787{,}105{,}577}{456{,}829{,}320{,}960}n+\frac {2{,}749{,}505{,}046{,}083}{153{,}494{,}651{,}842{,}560}}{n^{4}-\frac{1}{24}n^{3}+\frac {685{,}893{,}605}{845{,}980{,}224}n^{2}-\frac{14{,}787{,}105{,}577}{456{,}829{,}320{,}960}n+\frac {2{,}749{,}505{,}046{,}083}{153{,}494{,}651{,}842{,}560}} \\ &=V_{n}. \end{aligned}$$

We here offer some numerical computations (see Table 1) to show the superiority of our sequences $(U_{n})_{n\geq1}$ and $(V_{n})_{n\geq1}$ over the sequence $(\lambda_{n})_{n\geq1}$.

**Table 1:** **Comparison among approximation formulas (** **5.6** **)-(** **5.8** **)**

***n***	$\boldsymbol {\frac{\lambda_{n}-n!}{n!}}$	$\boldsymbol {\frac{U_{n}-n!}{n!}}$	$\boldsymbol {\frac{n!-V_{n}}{n!}}$
10	1.7686 × 10^−9^	3.6355 × 10^−11^	3.5843 × 10^−13^
100	1.7855 × 10^−14^	3.7108 × 10^−18^	3.7317 × 10^−22^
1,000	1.7857 × 10^−19^	3.7115 × 10^−25^	3.7332 × 10^−31^
10,000	1.7857 × 10^−24^	3.7115 × 10^−32^	3.7333 × 10^−40^

## References

[1] Abramowitz M, Stegun IA (1972). Handbook of Mathematical Functions with Formulas, Graphs, and Mathematical Tables.

[2] Copson ET (1965). Asymptotic Expansions.

[3] Alzer H (2003). On Ramanujan’s double inequality for the gamma function. Bull. Lond. Math. Soc..

[4] Batir N (2008). Sharp inequalities for factorial *n*. Proyecciones.

[5] Batir N (2010). Very accurate approximations for the factorial function. J. Math. Inequal..

[6] Batir N (2011). Improving Stirling’s formula. Math. Commun..

[7] Burić T, Elezović N (2011). New asymptotic expansions of the gamma function and improvements of Stirling’s type formulas. J. Comput. Anal. Appl..

[8] Burnside W (1917). A rapidly convergent series for $\log N!$. Messenger Math..

[9] Chen CP (2013). Unified treatment of several asymptotic formulas for the gamma function. Numer. Algorithms.

[10] Chen CP (2015). Inequalities and asymptotic expansions associated with the Ramanujan and Nemes formulas for the gamma function. Appl. Math. Comput..

[11] Chen CP (2014). Asymptotic expansions of the gamma function related to Windschitl’s formula. Appl. Math. Comput..

[12] Chen CP, Batir N (2012). Some inequalities and monotonicity properties associated with the gamma and psi functions. Appl. Math. Comput..

[13] Chen CP, Lin L (2012). Remarks on asymptotic expansions for the gamma function. Appl. Math. Lett..

[14] Chen CP, Liu JY (2015). Complete monotonicity properties and asymptotic expansions of the logarithm of the gamma function. Math. Inequal. Appl..

[15] Chen CP, Liu JY (2015). Inequalities and asymptotic expansions for the gamma function. J. Number Theory.

[16] Chen CP, Paris RB (2015). Inequalities, asymptotic expansions and completely monotonic functions related to the gamma function. Appl. Math. Comput..

[17] Chen CP (2016). A sharp version of Ramanujan’s inequality for the factorial function. Ramanujan J..

[18] Chen CP (2016). Monotonicity properties, inequalities and asymptotic expansions associated with the gamma function. Appl. Math. Comput..

[19] Gosper RW (1978). Decision procedure for indefinite hypergeometric summation. Proc. Natl. Acad. Sci. USA.

[20] Hirschhorn MD (2006). A new version of Stirling’s formula. Math. Gaz..

[21] Hirschhorn MD, Villarino MB (2014). A refinement of Ramanujan’s factorial approximation. Ramanujan J..

[22] Karatsuba EA (2001). On the asymptotic representation of the Euler gamma function by Ramanujan. J. Comput. Appl. Math..

[23] Lin L (2014). On Stirling’s formula remainder. Appl. Math. Comput..

[24] Lin L, Chen CP (2015). Asymptotic formulas for the gamma function by Gosper. J. Math. Inequal..

[25] Lu D (2014). A generated approximation related to Burnside’s formula. J. Number Theory.

[26] Lu D, Feng J, Ma C (2014). A general asymptotic formula of the gamma function based on the Burnside’s formula. J. Number Theory.

[27] Lu D, Song L, Ma C (2014). A generated approximation of the gamma function related to Windschitl’s formula. J. Number Theory.

[28] Lu D, Song L, Ma C (2015). Some new asymptotic approximations of the gamma function based on Nemes’ formula, Ramanujan’s formula and Burnside’s formula. Appl. Math. Comput..

[29] Lu D, Wang X (2013). A generated approximation related to Gosper’s formula and Ramanujan’s formula. J. Math. Anal. Appl..

[30] Lu D, Wang X (2014). A new asymptotic expansion and some inequalities for the gamma function. J. Number Theory.

[31] Mahmoud M, Alghamdi MA, Agarwal RP (2012). New upper bounds of *n*!. J. Inequal. Appl..

[32] Mortici C (2009). An ultimate extremely accurate formula for approximation of the factorial function. Arch. Math. (Basel).

[33] Mortici C (2010). Sharp inequalities related to Gosper’s formula. C. R. Math. Acad. Sci. Paris.

[34] Mortici C (2010). Product approximations via asymptotic integration. Am. Math. Mon..

[35] Mortici C (2010). New improvements of the Stirling formula. Appl. Math. Comput..

[36] Mortici C (2010). The asymptotic series of the generalized Stirling formula. Comput. Math. Appl..

[37] Mortici C (2010). Asymptotic expansions of the generalized Stirling approximations. Math. Comput. Model..

[38] Mortici C (2010). A class of integral approximations for the factorial function. Comput. Math. Appl..

[39] Mortici C (2010). Ramanujan formula for the generalized Stirling approximation. Appl. Math. Comput..

[40] Mortici C (2010). Best estimates of the generalized Stirling formula. Appl. Math. Comput..

[41] Mortici C (2011). On the gamma function approximation by Burnside. Appl. Math. E-Notes.

[42] Mortici C (2011). On Gospers formula for the Gamma function. J. Math. Inequal..

[43] Mortici C (2011). Improved asymptotic formulas for the gamma function. Comput. Math. Appl..

[44] Mortici C (2015). A new fast asymptotic series for the gamma function. Ramanujan J..

[45] Mortici C (2012). An improvement of the Ramanujan formula for approximation of the Euler gamma function. Carpath. J. Math..

[46] Mortici C (2011). Ramanujan’s estimate for the gamma function via monotonicity arguments. Ramanujan J..

[47] Mortici C (2011). A new Stirling series as continued fraction. Numer. Algorithms.

[48] Mortici C (2011). On Ramanujan’s large argument formula for the gamma function. Ramanujan J..

[49] Mortici C (2013). A continued fraction approximation of the gamma function. J. Math. Anal. Appl..

[50] Mortici C (2010). Very accurate estimates of the polygamma functions. Asymptot. Anal..

[51] Mortici C (2011). A new Stirling series as continued fraction. Numer. Algorithms.

[52] Nemes G (2010). Asymptotic expansion for $\log n!$ in terms of the reciprocal of a triangular number. Acta Math. Hung..

[53] Nemes G (2011). More accurate approximations for the gamma function. Thai J. Math..

[54] Nemes G (2010). New asymptotic expansion for the Gamma function. Arch. Math. (Basel).

[55] Alzer H (1997). On some inequalities for the gamma and psi functions. Math. Comput..

[56] Koumandos S (2006). Remarks on some completely monotonic functions. J. Math. Anal. Appl..

[57] Koumandos S, Pedersen HL (2009). Completely monotonic functions of positive order and asymptotic expansions of the logarithm of Barnes double gamma function and Euler’s gamma function. J. Math. Anal. Appl..

[58] Bercu G (2016). Padé approximant related to remarkable inequalities involving trigonometric functions. J. Inequal. Appl..

[59] Bercu G (2017). The natural approach of trigonometric inequalities-Pade approximant. J. Math. Inequal..

[60] Bercu G, Wu S (2016). Refinements of certain hyperbolic inequalities via the Padé approximation method. J. Nonlinear Sci. Appl..

[61] Brezinski C, Redivo-Zaglia M (2015). New representations of Padé, Padé-type, and partial Padé approximants. J. Comput. Appl. Math..

[62] Ramanujan S (1988). The Lost Notebook and Other Unpublished Papers.

